# Fuchs’ uveitis syndrome: a 20-year experience in 466 patients

**DOI:** 10.1038/s41598-024-59393-w

**Published:** 2024-04-14

**Authors:** Farzan Kianersi, Hamidreza Kianersi, Mohsen Pourazizi, Afsaneh Naderi Beni, Pegah Noorshargh

**Affiliations:** 1 Isfahan Eye Research Center, Department of Ophthalmology, Isfahan University of Medical Sciences, Isfahan, Iran; 2 Department of Ophthalmology, Feiz Hospital, Modares St., Qods Square, Isfahan, Iran

**Keywords:** Iranian population, Clinical characteristics, Fuchs uveitis syndrome, Epidemiological features, Ophthalmological findings, Diseases, Rheumatology, Signs and symptoms

## Abstract

Fuchs Uveitis Syndrome (FUS), also known as Fuchs Heterochromic Iridocyclitis, is a chronic form of uveitis characterized by mild inflammation primarily affecting one eye. This study aimed to investigate the clinical and epidemiological features of FUS in an Iranian population. A retrospective analysis was conducted on 466 patients diagnosed with FUS at an ophthalmology center affiliated with Isfahan University of Medical Sciences between 2003 and 2021. The Kimura et al. criteria were used for FUS diagnosis. Demographic data, clinical characteristics, misdiagnosed cases, concurrent diseases, and associated ocular findings were analyzed. The study included 507 eyes of 466 FUS patients, with a mean age of 34.01 ± 11.25 years. Iris atrophy, keratic precipitates, and vitritis were common clinical findings. Heterochromia was an infrequent feature. Initial misdiagnosis occurred in 13 patients, with pars planitis being the most common incorrect diagnosis. Toxoplasmosis and multiple sclerosis were common concurrent diseases. Pediatric FUS cases were noted, possibly attributed to early-onset manifestations. Differences in clinical characteristics were observed when compared to other populations. This study provides insights into the clinical and epidemiological aspects of FUS in an Iranian population. Variations in clinical features, misdiagnosis patterns, and concurrent diseases were noted. Attention to specific clinical parameters can aid in accurate FUS diagnosis. Understanding these differences contributes to a better understanding of FUS presentation and its relationship with other diseases.

## Introduction

Fuchs uveitis syndrome (FUS) also known as Fuchs heterochromic iridocyclitis, is a chronic form of uveitis characterized by mild inflammation typically in one eye, often asymptomatic during routine ocular examinations^1,2^.

FUS ranks as the second most common non-infectious uveitis in some reports^3^. While the incidence of FUS spans between 1.8 and 22.7% in developed countries, it remains lower, ranging from 0 to 5.6%, in developing nations^4,5^. Certain regions report FUS patients constituting up to 22.7% of overall uveitis cases, or up to 45% when restricted to anterior uveitis instances^6^. In our earlier investigation at an Iranian Tertiary Eye Center, FUS accounted for over a third of anterior uveitis cases^7^.

Classic clinical manifestations of FUS encompass heterochromia, keratic precipitates (KP), mild iridocyclitis, and iris atrophy without posterior synechiae or cystoid macular edema, although chronic inflammation persists. Patients commonly report heterochromia in the affected eye, and vision changes are typically attributed to secondary complications such as cataracts and glaucoma^2,8–10^.

While the exact cause of FUS remains elusive, diagnostic reliance remains on clinical assessment, despite the appeal of a single etiological agent and a sensitive laboratory test^2,11^. Over time, numerous proposed etiological theories have been discredited, with infectious theories such as CMV and Rubella virus persisting as a plausible cause^12^.

Previous studies have suggested variations in the clinical spectrum of FUS in different populations^3,13–15^. Limited clinical data on unique FUS patterns, including pediatric FUS, bilateral cases, and misdiagnosed instances, have been documented^16–19^.

Epidemiologic studies with a relatively considerable number of patients for FUS in Iran are scarce^7,18^. To the best of our knowledge, the present study is one of the few large-scale studies on various clinical and epidemiological features of FUS. The present study aimed to draw attention to various clinical and epidemiological features of FUS in Iran.

## Materials and methods

### Patients and setting

This was a retrospective study of patients with a diagnosis of FUS at the referral outpatient clinic of Uveitis in a referral ophthalmology center affiliated to Isfahan University of Medical Sciences, Isfahan during 2003–2021. The study protocol was sanctioned by the Ethics Committee of Isfahan University of Medical Sciences, Iran (Code: IR.MUI.MED.REC.1398.72) and all methods were performed in accordance with the relevant guidelines and regulations. Informed consent has been taken from patients to use their data for research purposes.

Eligibility criteria encompassed patients definitively diagnosed with FUS, each with at least a 2-year follow-up period. The Kimura et al. criteria were employed for FUS diagnosis, encompassing specific ocular findings^1,11,20^.

Exclusion criteria were lack of diagnosis of FUS in the medical record on discharge, confirmation of an alternate diagnosis, incompatible clinical assessment, doubtful diagnosis, and insufficient information.

### Data collection

Data from medical records of all subjects including patients’ sex, age, previous medical history, drug history, clinical and ocular symptoms, presence of any systemic diseases, management strategies, and clinical course were reviewed. Ophthalmological data consisted of slit-lamp biomicroscopy findings, Goldmann applanation tonometry, and indirect ophthalmoscopy findings. As warranted, additional tests were employed to assess underlying diseases.

### Statistical methods

Frequency distribution tables were used to report categorical variables, and numerical variables were described with median and range. The relationship between each categorical variable and age group and sex was assessed by Chi-square test or Fisher exact test. Statistical analysis was performed with SPSS-18 software at a 95% confidence level.

### Ethics approval

The protocol for this study was approved by the Ethics Committee of Isfahan University of Medical Sciences, Isfahan, Iran (Code: IR.MUI.MED.REC.1398.72).

## Results

Five hundred and seven eyes from 466 patients with a final diagnosis of FUS were included in the study. Forty-one patients (9.8%) had bilateral FUS. The mean age of the patients was 34.01 ± 11.25 years and there were 243 females (52.1%). The most common chief complaints were blurred vision (70.4%), floaters (15.6%), and incidental (10.3%). Iris atrophy, small- to medium-sized stellate KPs, and vitritis were noted in 497 (98%), 496 (97.8%), and 408 (80.5%) eyes, respectively. Iris heterochromia was observed in 40 (7.9%) eyes. Reversal of iris heterochromia was seen in one patient with blue eyes. Sixty-five patients had raised IOP (12.8%), of which 6 patients needed glaucoma surgery. Cataract and history of cataract surgery were recorded in 221 (43.6%) and 124 (24.5%) eyes, respectively (Table 1).

**Table 1:** Comparison of demographic and clinical characteristics between patients/eyes correctly diagnosed as FU and patients/eyes initially misdiagnosed as other uveitis.

Variables	FUS with the correct diagnosis (507 eyes of 466 patients)	FUS with the misdiagnosis (18 eyes of 13 patients)	*P*-value
Age (years)			
Mean ± SD	34.01 ± 11.25	29.00 ± 9.79	0.07
Median [min–max]	32 [2–75]	28 [15–50]	
Gender f (%)			
Male	223 (47.9)	5 (38.5)	0.58
Female	243 (52.1)	8 (61.5)	
Laterality f (%)			
Unilateral	425 (91.2)	8 (61.5)	0.005
Bilateral	41 (9.8)	5 (38.5)	
Eye f (%)			
Right	241 (47.5)	7 (38.9)	0.63
Left	266 (52.5)	11 (61.1)	
Slit lamp biomicroscopy f (%)			
KPs	496 (97.8)	18 (100)	0.99
AC cell	298 (58.5)	8 (44.4)	0.23
Iris atrophy/heterochromia	497 (98)	4 (22.2)	< 0.001
Iris nodule	124 (24.5)	3 (16.7)	0.34
Vitritis	408 (80.5)	15 (83.3)	0.99
Vitreous opacity and strands	280 (55.2)	14 (77.8)	0.08
Glaucoma	65 (12.8)	6 (33.3)	0.02
Lens status f (%)			
Cataract	221 (43.5)	6 (33.3)	0.003
PC-IOL	124 (24.5)	0 (0)	
No significant cataract	162 (32)	12 (66.7)	

*P-value resulted from Mann–Whitney *U* test or Chi-squared (or Fisher exact test) for between groups comparisons.

*KP* keratic precipitate. *PCIOL* posterior chamber intraocular lens, *AC* anterior chamber.

Eighteen eyes of 13 patients had initially misdiagnosed as other uveitis. The initial diagnosis of these patients before follow up were pars planitis (PP) (8/13), toxoplasmosis (2/13), posterior scleritis (1/13), Posner–Schlossmann syndrome (1/13), and undiagnosed case (1/13). Table 1 presents a comparison of demographic and clinical characteristics between patients/eyes correctly diagnosed as FUS and patients/eyes initially misdiagnosed as other uveitis. Bilateral involvement was more common in the group with an initially wrong diagnosis (P = 0.005). Iris atrophy and cataract in the misdiagnosed group had a lower rate compared to the corrected diagnosis group (P < 0.001 and P = 0.003, respectively). In addition, patients in the misdiagnosed group had a higher rate of glaucoma (P = 0.02) (Table 1).

Comparison of clinical findings between pediatrics/adults and male/female are summarized in Table 2. Iris atrophy was more common in adult patients (98.3% in adults versus. 92% in pediatrics; P = 0.08).

**Table 2:** Comparison of clinical findings between pediatrics/adults and male/female in patients with FUS.

Variables	Age grouping			Gender		
	Pediatrics	Adults	*P*-value	Male	Female	*P*-value
Eye f (%)						
Right	13 (52)	228 (47.3)	0.68	127 (52.9)	114 (47.3)	0.02
Left	12 (48)	254 (52.7)		113 (47.1)	153 (52.7)	
Slit lamp biomicroscopy f (%)						
KPs	25 (100)	471 (97.7)	0.99	237 (98.8)	259 (97)	0.23
AC cell	15 (60)	283 (58.7)	0.99	146 (60.8)	152 (56.9)	0.41
Iris atrophy/heterochromia	23 (92)	474 (98.3)	0.08	233 (97.1)	264 (98.9)	0.20
Iris nodule	8 (32)	116 (24.1)	0.34	58 (24.2)	66 (24.7)	0.92
Vitritis	18 (72)	390 (80.9)	0.29	193 (80.4)	215 (80.5)	0.99
Vitreous opacity and strands	10 (40)	270 (56.1)	0.14	134 (55.8)	146 (54.9)	0.85
Glaucoma	5 (20)	60 (12.4)	0.35	29 (12.1)	36 (13.5)	0.69
Lens status f (%)						
Cataract	10 (40)	120 (24.9)	0.16	99 (41.3)	122 (45.7)	0.72
PC-IOL	4 (16)	211 (43.8)		60 (25)	64 (24)	
No significant cataract	11 (44)	151 (31.3)		81 (33.7)	81 (30.3)	

*P-value resulted from Chi-squared (or Fisher exact test) for between groups comparisons.

*KP* keratic precipitate, *PCIOL* posterior chamber intraocular lens, *AC* anterior chamber.

Eight patients with FUS were misdiagnosed as having Behcet disease (BD) and were on immunosuppressive therapy when referred to our referral center. Table 3 presents concurrent findings and diseases in FUS patients. The most common concurrent diseases were toxoplasmosis (30 eyes of 18 patients) and multiple sclerosis (6 patients). The most common concurrent ocular findings were epiretinal membrane (15 patients), amblyopia (12 patients), and retinal detachment (RD) (5 patients) (Table 3).

**Table 3:** Concurrent findings and diseases in FUS patients.

Toxoplasmosis scar	18 patients	Twelve patients had bilateral involvement One patient had macular coloboma
Epiretinal membrane in examination	15 patients	
Amblyopia	12 patients	Six cases in contralateral eyes Ten cases had anisometropic amblyopia and two cases had strabismus amblyopia
Multiple sclerosis	6 patients	All unilateral involvement
Retinal detachment	5 patients	Three patients one week after cataract surgery, had retinal detachment. Two patients were phakic
Retinal break	5 patients	Without any retinal detachment
Central serous chorioretinopathy	3 patients	One patient had CSCR in contralateral eyes
Keratoconus	2 patients	
Hypothyroidism	2 patients	
Retinitis pigmentosa	1 patient	
Breast cancer	1 patient	
Testicular cancer	1 patient	
Herpetic anterior uveitis	1 patient	The patient presented with FUS in contralateral eye
Recurrent anterior uveitis	1 patient	The patient presented with FUS in contralateral eye
Hypophyseal adenoma	1 patient	
Oculocutaneous albinism	1 patient	
Urbach–Wiethe syndrome	1 patient	Patient had bilateral FUS
Niemann pick type-C	1 patient	

## Discussion

The result of our study identified several differences between the FUS in our population compared to others. Iris heterochromia was an uncommon clinical feature and PP was the most common causes of mistaken diagnosis in our study. The most common concurrent diseases in our study were toxoplasmosis and multiple sclerosis (MS).

Differences in the clinico-epidemiological pattern of FUS can be attributed to geographic, ethnicity, and genetic/epigenetic factors. These findings can provide new insights into the clinical and epidemiological aspects of FUS.

Although there are some differences between the results of our study and previous studies, major demographic data and most clinical characteristic of FUS were similar to other studies^14,15,21–23^. Consistent with the previous study in another Iranian population^18^, in our study iris heterochromia was an uncommon clinical feature. In contrast, studies from European countries showed that heterochromia is a more common finding in FUS^24–26^. A possible explanation for this contrast can be attributed to oculocutaneous phenotype. In fair phenotype due to pigmentary dilution of iris, the pigmentary changes related to the FUS may be more apparent.

FUS varies widely in differential diagnosis and can be considered as a great imitator in differential diagnosis of uveitis. FUS may be misdiagnosed due to its similarity to other inflammatory conditions or uveitis. FUS should be considered in all patients with uveitis, especially in patients with a history of uncertain clinical criteria for other differential diagnoses. Similar to previous studies, PP was the most common cause of misdiagnosis in our study^18,27,28^. Existence of iris changes, bilateral involvement, cataract, and glaucoma were four factors that differed between accurate and mistaken diagnoses of FUS in our study. In challenging cases, attention to changes in iris pigment, unilateral involvement, and existence of cataract can be helpful parameter for true diagnosis of FUS.

In our study, there is a possible important linkage between FUS with Toxoplasmosis. Many authors have looked for a link between toxoplasmosis and FUS^29^. For example in the study of Toledo de Abreu et al. association of FUS with ocular toxoplasmosis was seen in 13 patients in FUS^30^.

It is unclear whether this relatively high co-incidence of FUS and these conditions are related to the high prevalence of toxoplasmosis in our area, up to 43% seroprevalence according to some reports^31^, or a direct association^32^. Although it cannot be excluded that in some patients with FUS, the observed Toxoplasmosis may represent a co-incidence of two diseases, there is some hypothesis for a potential association between Toxoplasmosis and FUS.

In addition, regarding a possible association between toxoplasmosis and FUS, six patients had FUS co-existing with MS. Regarding the high prevalence of MS in our area, Isfahan, Iran^33,34^, further investigation is necessary for the identification of possible association of MS and FUS and possible etiopathogenesis pathway or incidental co-occurrence.

The frequency of RD in FUS is not well defined. Five patients in our study had RD. Severe inflammation of the vitreous may lead to vitreous traction causing the tractional and rhegmatogenous retinal detachment^35^.

FUS is a great imitator and a wide range of differential diagnoses should be considered for accurate diagnosis. In our study 8 patients were referred to us with clinical impression of BD, while on exact ocular examination and re-checking of the clinical criteria, the primary diagnoses of BD were ruled out, and patients were considered as FUS. On the other hand, eight patients had diagnoses of FUS and with long-term follow-up, alternative diagnoses were confirmed.

FUS is a disease of young adults and childhood FUS is a rare condition^17^. In our study, 25 patients had childhood FUS. On one hand, the relatively higher patient count can be ascribed to a selection bias stemming from the referral center's focus on uveitis. On the other hand, FUS might initiate during early childhood, yet its clinical manifestations might not manifest at the disease's outset. Consequently, diagnosis could potentially be postponed for several years^17^.

Although our study had some limitations including the retrospective nature of the study, current study, which included 507 eyes from 466 patients, can provide some references for the difference of FUS between Iranian subjects and others. Note that the number of cases in our study in comparison with others is considerable.

## Conclusion

The current study demonstrated several differences between Iranian FUS patients and others, including clinical features, misdiagnosis patterns, and concurrent diseases. In challenging cases, attention to changes in iris pigment, unilateral involvement, and existence of cataract can be helpful parameter for the true diagnosis of FUS.

## Data Availability

The datasets generated during and/or analyzed during the current study are available from the corresponding author on reasonable request.

## Abbreviations

FUS: Fuchs’ uveitis syndrome
KP: Keratic precipitates
BD: Behcet disease
MS: Multiple sclerosis
PP: Pars planitis
